# *Contrast Media & Molecular Imaging*, a Journal at the Crossroads of the Scientific and Medical Disciplines of Biomedical Imaging

**DOI:** 10.1155/2019/7213829

**Published:** 2019-12-09

**Authors:** Luc Zimmer

**Affiliations:** ^1^Université de Lyon, INSERM, CNRS, Lyon Neuroscience Research Center, Lyon, France; ^2^Hospices Civils de Lyon, CERMEP-Imaging Platform, Lyon, France; ^3^National Institute for Nuclear Science and Technology, Saclay, Paris, France

I am very honoured to take on the role of Chief Editor of *Contrast Media & Molecular Imaging* (CMMI).

CMMI was founded in 2006, originally published by John Wiley & Sons. On 1 January 2017, the journal became fully Open Access, thanks to a publishing partnership between John Wiley & Sons and Hindawi Limited. CMMI was initially intended for a readership of chemists and biochemists developing contrast agents, particularly in MRI, fully justifying the specific reference to “Contrast Media” in its title. Professor Silvio Aime of the University of Torino and Professor Robert N. Muller of the Université de Mons, both eminent specialists in chemistry applied to molecular MRI, were its first Chief Editors.

MRI has benefited from very rapid progress in both instrumentation (such as scanners with increasingly high magnetic fields and new acquisition sequences) and emerging contrast agents. Biomedical imaging enables noninvasive exploration of animal models, but its main purpose for application in humans is to help in diagnosis or with therapeutic strategies. This potential for back-and-forth discussion between preclinical and clinical research gave biomedical imaging a unique place, long before the expression “translational research” became fashionable in other fields.

MRI has also gone beyond its initial field of anatomic imaging, with the rapid development of functional MRI and tractography and the emergence of contrast agents opening up new ways of visualizing targets of interest for physiology and pathophysiology. MRI can now claim to “see” almost all biological tissues and to follow some of their physiological functions at the molecular, anatomical, or functional level. In this context, the reference to “Molecular Imaging” takes on its full meaning in the title of the journal. The challenge for these contrast agents now is to continue to enhance their sensitivity, with the aim of injecting ever smaller quantities which are less prone to side effects and, for new contrast agents, to eventually merit the status of diagnostic drugs.

CMMI initially focused on MRI, but it is now time to decompartmentalize its audience. The biomedical MRI technologist, scientist, and clinician communities have begun to discuss and collaborate with their nuclear medicine colleagues. Nuclear medicine has a long history in the fields of radiolabelled contrast agents, also known as radiopharmaceuticals. This scientific and medical community—which I belong to as a radiopharmacist and pharmacologist—has extensive experience in translational research and the development of contrast agents, focusing on those specific properties with particular importance to the community, such as the concept of a “tracer dose.” Forging links—or highlighting different approaches—between the contrast agent communities in MRI and nuclear medicine is of great interest and a source of joint progress. This is all the more essential now that the introduction of hybrid scanners is leading to the use of contrast agents that are also hybrid, i.e., detected by both MRI cameras and PET or SPECT cameras.

The physics and biophysics underlying MRI and PET detection clearly have their own specific requirements, and the underlying chemistry of contrast agents differs between a gadolinium-chelated conjugated agent and a small heterocyclic molecule radiolabelled with fluorine 18. Despite this, there are many common aspects in the biology of these agents for imaging, from the idea of bioavailability to certain pharmacokinetic parameters, and from the idea of specificity to that of sensitivity.

In addition, it should not be forgotten that biomedical diagnostic imaging has made great progress alongside other technologies. Radiographic imaging benefited from the development of iodine molecules several decades ago. The recent emergence of spectral photon-counting CT has opened up the field to new imaging possibilities with its own contrast agents. Here too, the underlying aim will be translation to humans once drug agency authorisations have been obtained. Finally, there is ultrasound imaging, which with its microbubble contrast agent may have more limited preclinical and clinical uses; however, the field is also set to develop in coming years, particularly in the recent field of ultra-fast ultrasound imaging.

In these continuously evolving fields, CMMI aims to be a vector for scientific exchange for the community of researchers who invent, design, develop, validate, and use these molecules, preclinically and clinically, for imaging.

*Luc Zimmer, PharmD, PhD is professor of pharmacology at the University Hospital of Lyon (Université Claude Bernard Lyon 1 & Hospices Civils de Lyon). He is also director of the CERMEP, an imaging platform dedicated to biomedical research, and head of a research team at the Lyon Neuroscience Research Center (CNRS-INSERM)*.

*Luc Zimmer*

